# The Impact of Age on the Accuracy of Sexual Dimorphism Using Anthropometric Indices of Maxillary Sinus 

**DOI:** 10.30476/dentjods.2025.104945.2561

**Published:** 2026-03-01

**Authors:** Najmeh Movahhedian, Leila Gharemani, Fatemeh Akbarizadeh, Leila Khojastepour

**Affiliations:** 1 Dept. of Oral and Maxillofacial Radiology, School of Dentistry, Shiraz University of Medical Sciences, Shiraz, Iran.; 2 Undergraduate Student, Student Research Committee, School of Dentistry, Shiraz University of Medical Sciences, Shiraz, Iran.; 3 Orthodontic Research Center, School of Dentistry, Shiraz University of Medical Sciences, Shiraz, Iran.; 4 Dept. of Oral and Maxillofacial Radiology, School of Dentistry, Shiraz University of Medical Sciences, Shiraz, Iran.

**Keywords:** Sexual Dimorphism, Anthropometry, Cone-Beam Computed Tomography, Maxillary Sinus

## Abstract

**Background:**

The potential benefit of maxillary sinus measurements for analysis of sexual dimorphism has been proved. Also, it has been shown that maxillary sinus dimensions, as the reliable sex indicators, diminish with age due to physiological and morphological alterations.

**Purpose:**

This study aimed to evaluate the effect of age on the accuracy of maxillary sinus indices for sex determination.

**Materials and Method:**

In this cross-sectional study’s research 240 cone beam computed tomography (CBCT) scans (120 males, 120 females), aged 20-70 years old, were evaluated retrospectively. Subjects were categorized into four age groups: 20-29, 30-39, 40-49, and ≥50 years. Each group consisted of 60 subjects. Maximum sinus height, width, length, and distance between two maxillary sinuses were evaluated.

**Results:**

All the measurements were higher in men than in women. Generally, the strongest sex indicator was maxillary sinus width However, when analyzing different age groups, the most reliable indicators for determining sex were the distance between the sinuses in the 20-29 age group, sinus height in the 40-49 group, and sinus width in both the 30-39 and 50-and-above age groups. The specific sex discriminant formula showed an accuracy of 78.3% for the ages of 20-29 and 40-49 years as well as 71.7% for the 30-39 and ≥50 age groups.

**Conclusion:**

The specific sex discriminant formula presented in this study showed noticeable accuracies for sex determination. Additionally, discriminant analysis revealed that the anthropometric measurements of the maxillary sinus exhibit varying degrees of sexual dimorphism across different age groups.

## Introduction

Currently, determining the sex of corpses remains a challenging issue in forensic medicine. Sex dimorphism yields reliable results by evaluating the pelvis, skull, and long bones [ 1 - 2 ]. Although in highly damaged skulls, the skeletal bones may be significantly affected, it has been noted that the maxillary sinuses often remain intact [ 3 - 6 ]. Moreover, the airway patterns within the sinuses are so unique that no two individuals have identical sinus airways [ 7 ]. 

Lifelong physiological and morphological changes occur in the human skull due to intrinsic and extrinsic factors [ 8-9 ]. Similarly, some studies have assessed the correlation between age and maxillary sinus dimensions. Velasco-Torres . [ 10 ] conducted a comprehensive analysis of this relationship. Their research demonstrated an indirect correlation between age and both sinus width (mediolateral dimension) and height (distance from the meatus to the sinus floor) in dentate patients, as well as between age and sinus volume in partially edentulous individuals. Ajiri . [ 11 ] also identified a direct correlation between maxillary sinus volume and age until the age of 20 years, after which sinus volume decreased with age (correlation coefficient: -0.43). Nowak . [ 12 ] also reported an inverse correlation between the maxillary sinus dimension and age after the age of 30.

Multiple investigations have evaluated the potential of maxillary sinus dimensions for sexual dimorphism analysis using both linear and volumetric measurements [ 3 , 6 , 13 - 18 ]. Despite these efforts, the influence of age on sex determination accuracy based on maxillary sinus morphology remains understudied. To our knowledge, only one published study [ 3 ] has systematically examined age as a confounding variable in this context. However, their research had notable methodological limitations; it omitted discriminant analysis (a robust multivariate statistical approach that enhances result reliability) and failed to propose a predictive formula for clinical application.

This study aims to (1) quantify age-related variations in the sexual dimorphism potential of maxillary sinus anthropometric indices using cone beam computed tomography (CBCT) and (2) establish an age-stratified discriminant formula to improve forensic and diagnostic sex determination accuracy.

## Materials and Method

This cross-sectional study’s research protocol received ethical approval from the Human Ethics Review Committee of the Faculty of Dentistry, University of Medical Sciences, Shiraz, Iran (#IR.SUMS.DENTAL.REC. 1399.087).

This study analyzed 240 CBCT scans from 120 males and 120 females aged 20–70 years. These radiographs were selected from the archive of oral and maxillofacial radiology department and were taken for purposes other than the present study. Participants under 20 years were excluded to avoid confounding effects from ongoing developmental changes of the maxillary sinuses. The patients were referred to the Oral and Maxillofacial Radiology Department at Shiraz Dental School between January 2018 and May 2021 for clinical indications unrelated to this research. All patients provided written informed consent at the time of imaging, permitting the anonymous use of their radiographic data for research purposes.

CBCT scans were selected for the study based on the following criteria: adequate image quality, a field of view encompassing both maxillary sinuses and the entire maxillary dental arch, and absence of artifacts. Scans were excluded if there was any positive history of trauma, fracture, prior surgical interventions, and congenital craniofacial anomalies including cleft lip and palate, or extraction of more than one maxillary posterior tooth (premolar or molar) as well as finding any sign of pathologies affecting the maxillary sinuses or jaws, except for mild mucositis.

Participants were stratified into four age categories: 20–29, 30–39, 40–49, and ≥50 years. Each group contained 60 individuals, with equal representation of males and females across all age and sex subgroups.

**Figure 1:**
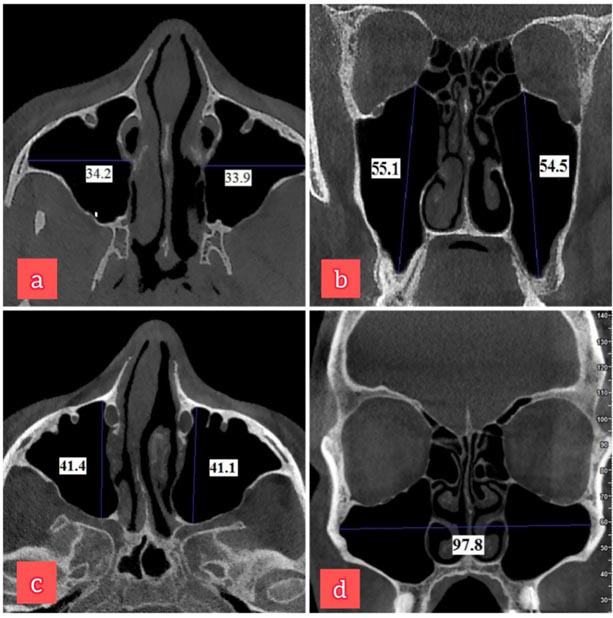
Shows the various measurements performed in this study: **a:** the maximum sinus width; **b:** The maximum sinus height; **c:** The maximum sinus length; **d:** The intermaxillary distance

The following anthropometric parameters were measured bilaterally on each CBCT scan (Figure 1) including (a) Maximum sinus width: the maximum perpendicular distance from the outermost point of the lateral wall of the maxillary sinus to the medial wall on the axial sections; (b) Maximum sinus height: the maximum distance between the uppermost and the lowermost points of the maxillary sinus borders on the coronal sections; (c) Maximum sinus length: the maximum distance between the most anterior and posterior points of the sinus walls on the axial sections, and additionally, (d) Maximum distance between the outermost borders of the right and left maxillary sinus walls on the coronal sections was measured in each scan. 

The measurements were performed on sections containing the most significant dimension within their respective planes. This determination was made after a comprehensive evaluation of all available slices. When dimensions were nearly equivalent across consecutive sections, measurements were systematically recorded across multiple slices to identify the maximum size.

All CBCT scans were acquired using the New Tom VGi evo CBCT unit (QR S.R.L. Company, Verona, Italy) with the following parameters: 3 mA tube current, 1.8 seconds exposure time, 110 kVp, and 0.3 mm voxel size. Patients were positioned in a standard orientation, with the occlusal plane parallel and the sagittal plane perpendicular to the floor. 

The measurements were done by a well-trained final-semester dental student and an oral and maxillofacial radiologist using NNT Viewer software (NNT V2.21, Image Works, Verona, Italy). One-third of the scans were re-evaluated after a one-month interval to assess inter- and intra-examiner reliability.

### Statistical analysis

To assess intra- and inter-observer reliability, intraclass correlation coefficients (ICCs) were calculated. Student’s t-test (two-tailed, significance threshold: *p*< 0.05) was used to compare variables between sexes. Discriminant analysis was performed to develop a sex-discrimination equation and evaluate prediction accuracy. All analyses were conducted using SPSS statistical software (version 26.0; IBM Corp., Armonk, NY, USA).

## Results

The ICC values, ranging from 0.90 to 0.97, demonstrated high inter-examiner and intra-examiner reliabilities for all variables. 

The mean age of the subjects, comprising 120 males and 120 females, was 39.7±11.9 years, with males averaging 39.3±11.5 years and females averaging 40±12.2 years.

According to the results of the current study, no significant differences were found between the measurements of the right and left maxillary sinuses in both sexes (all *p*> 0.05). 

Based on the result of the t-test, significant differences were observed between men and women in all the maxillary sinus measurements regardless of age; all the measures were statistically higher in men (all *p*< 0.001) (Table 1). When considering the age groups, almost all the measurements were statistically higher in men except for the width measurements of the 30-39-year-old age group (*p*= 0.139 and 0.127, respectively, for the right and left maxillary sinus) and the length measurement of the left maxillary sinus in 40-49-year-old age group (*p*= 0.062) (Table 2).

**Table 1:** Comparison of the mean ± standard deviation (SD) of each maxillary sinus parameter between two sexes

Parameter		Male	Female	*p* Value*
		Mean±SD	Mean±SD	
Maxillary sinus height	R	41.5 ± 6.3	35.5 ± 5.2	< 0.001
	L	41.7 ± 5.7	36.4 ± 4.7	< 0.001
Maxillary sinus length	R	40.8 ± 4.1	38.2 ± 3.6	< 0.001
	L	41.0 ± 3.5	38.2 ± 3.6	< 0.001
Maxillary sinus width	R	26.9 ± 5.4	24.1 ± 4.4	< 0.001
	L	27.0 ± 5.2	24.6 ± 4.2	< 0.001
Intermaxillary distance		84.6 ± 7.8	78.7 ± 6.8	< 0.001

R: right; L: left

*Paired t-test, *p*< 0.05 was considered significant

**Table 2:** Comparison of the mean ± standard deviation (SD) of each parameter between two sexes in different age groups

Age group	Parameter		Male	Female	*p* Value*
			Mean±SD	Mean±SD	
20-29 years	Maximum height	R	42.6 ± 5.8	36.9 ± 4.6	<0.001
		L	42.7 ± 5.8	37.8 ± 4.5	0.001
	Maximum width	R	28.5 ± 5.4	25.0 ± 3.7	0.006
		L	28.7 ± 5.0	25.6 ± 4.0	0.010
	Maximum length	R	41.7 ± 3.9	39.5 ± 2.6	0.011
		L	41.9 ± 3.7	39.3 ± 2.8	0.003
	Intermaxillary distance		86.9 ± 7.8	80.0 ± 6.1	<0.001
30-39 years	Maximum height	R	41.1 ± 7.0	35.1 ± 5.5	0.001
		L	41.5 ± 6.3	35.7± 4.8	<0.001
	Maximum width	R	25.9 ± 5.9	23.7 ± 5.0	0.139
		L	26.4 ± 5.3	24.5 ± 4.1	0.127
	Maximum length	R	41.3 ± 4.6	38.1 ± 4.0	0.006
		L	41.4 ± 3.9	37.7 ± 3.2	<0.001
	Intermaxillary distance		83.4 ± 8.2	78.7 ± 6.5	0.018
40-49 years	Maximum height	R	42.4 ± 6.0	35.9 ± 6.1	<0.001
		L	41.9 ± 5.9	37.3 ± 4.9	0.002
	Maximum width	R	27.1 ± 5.3	24.3 ± 4.1	0.029
		L	27.3 ± 5.8	24.6 ± 4.1	0.040
	Maximum length	R	40.4 ± 3.2	38.6 ± 3.4	0.035
		L	40.5 ± 3.0	38.9 ± 3.6	0.062
	Intermaxillary distance		84.1 ± 8.2	78.9 ± 7.3	0.012
≥50 years	Maximum height	R	39.9±6.1	34.2±4.6	<0.001
		L	40.7±4.8	34.8±4.2	<0.001
	Maximum width	R	26.3±4.6	23.3±4.7	0.016
		L	25.9±3.9	23.5±4.7	0.038
	Maximum length	R	39.8±4.6	36.7±3.9	0.007
		L	40.1±3.2	36.8 ±4.2	0.001
	Intermaxillary distance		84.0± 6.9	77.1 ±7.2	<0.001

R: right; L: left

*Independent t-test; *p*< 0.05 was considered significant

Table 3 compares the parameters across different sex and age groups. Based on these results, age did not have any statistically significant effect on the maxillary sinus indices of both sexes except for the length measurements in females. The length measurements were significantly higher in the 20-29-year-old age group compared to the ≥ 50-year-old age group for both the right (*p*= 0.018) and left (*p*= 0.036) sinuses. 

**Table 3:** Comparison of the mean values of each parameter between the different age groups in both sexes

Parameter	Side	Sex	Age					
			20-29, 30-39	20-29, 40-49	20-29, ≥50	30-39, 40-49	30-39, ≥50	40-49, ≥50
Maxillary sinus height	R	M	1.000	1.000	0.559	1.000	1.000	0.578
		F	1.000	1.000	0.284	1.000	1.000	1.000
	L	M	1.000	1.000	1.000	1.000	1.000	1.000
		F	0.496	1.000	0.088	1.000	1.000	0.256
Maxillary sinus length	R	M	1.000	1.000	0.424	1.000	1.000	1.000
		F	0.884	1.000	0.018*	1.000	0.670	0.242
	L	M	1.000	1.000	0.252	1.000	1.000	1.000
		F	0.512	1.000	0.036*	1.000	1.000	0.121
Maxillary sinus width	R	M	0.345	1.000	0.690	1.000	1.000	1.000
		F	1.000	1.000	0.836	1.000	1.000	1.000
	L	M	0.479	1.000	0.193	1.000	1.000	1.000
		F	1.000	1.000	0.319	1.000	1.000	1.000
Intermaxillary distance		M	0.389	1.000	0.882	1.000	1.000	1.000
		F	1.000	1.000	0.668	1.000	1.000	1.000

R: Right; L: Left; M: Male; F: Female

* Independent t-test; *p*< 0.05 was considered significant

Discriminant analysis revealed that the strongest indicator for sex estimation was maxillary sinus width, followed in descending order of predictive strength by maxillary sinus height, inter-sinus distance, and maxillary sinus length. Through evaluation of Canonical Discriminant Function Coefficient, the discriminant function for all the measurements, without considering the age groups, was formulated as follows: 

D = -11.901 + 0.114 (distance) + 0.199 (height) + 0.031 (length) - 0.247(width)

The D-score for differentiating between the sexes is 0. The D-scores above 0 define men, and the ones below represent women.

The discriminant analysis concerning the age groups was also done, and the results are presented in Table 4. As the Standardized Canonical Discriminant Function Coefficients show, the most accurate parameter for sex determination in the 20-29 age group was the distance between maxillary sinuses, in the 30-39 and ≥50 age group was the maximum sinus width, and in the 40-49 age group was the maximum sinus height. 

**Table 4:** The results of discriminant analysis for all the measurements in each age group

		C.D.F.C*	S.D.F.C **
20-29 years	Maximum height	0.168	0.841
	Maximum width	-0.291	-1.296
	Maximum length	0.026	0.085
	Intermaxillary distance	0.186	1.306
✰ D2= -15.466+0.186(distance)+0.168(height)+0.026 (length)- 0.291(width)			
30-39 years	Maximum height	0.192	1.097
	Maximum width	-0.369	-1.771
	Maximum length	0.096	0.361
	Intermaxillary distance	0.145	1.073
✰ D3= -13.646+0.145(distance)+0.192(height)+ 0.096 (length) – 0.369(width)			
40-49 years	Maximum height	0.219	1.231
	Maximum width	-0.063	-0.292
	Maximum length	-0.049	-0.154
	Intermaxillary distance	0.011	0.082
✰ D4= -5.913 + 0.11(distance) + 0.219(height) -0.049(length) – 0.063(width)			
≥50 years	Maximum height	0.219	1.053
	Maximum width	-0.315	-1.323
	Maximum length	0.052	0.195
	Intermaxillary distance	0.132	0.935
✰ D5=-13.048+0.132(distance)+0.219(height)+0.052 (length) – 0.315(width)			

* Canonical Discriminant Function Coefficients

** Standardized Canonical Discriminant Function Coefficients

✰ The discrimination score for differentiating between two sexes was 0 for all the age groups (values above 0 define men, and values below 0 define women).

The discriminant functions for each age group are also detailed in Table 4. The accuracy of sex determination in this study was 71.7% for males and 75% for females, with an overall accuracy of 73.3% (Table 5). 

**Table 5:** The accuracy rate of sex prediction based on age groups and sexes

Age group	Accuracy rate %		
	Male	Female	Total
20-29	76.7	80.0	78.3
30-39	66.7	76.7	71.7
40-49	66.7	76.7	71.7
≥50	73.3	83.3	78.3

## Discussion

It has been demonstrated that establishing population-specific anthropometric standards for human sexual dimorphism is an essential step in forensic identification [ 16 , 19 - 21 ]. In this study, we assessed the influence of age on the accuracy of using maxillary sinus anthropometric indices for determining sexual dimorphism in a sample of the Iranian population. 

We used CBCT images to conduct sinus measurements. Compared to multi-slice computerized tomography, CBCT offers several advantages in forensic investigations, including its compact size, portability, and cost-effectiveness. It also imposes a lower absorbed radiation dose on the patients while providing accurate images of craniofacial bones with sub-millimeter resolution [ 22 - 23 ]. Additionally, the validity and accuracy of CBCT for maxillary sinus measurements have been previously verified [ 24 ]. 

It is documented that maxillary sinuses undergo physiological pneumatization until skeletal development concludes [ 25 ] or around 20 years [ 11 ]. To ensure methodological reliability, individuals below 20 years were excluded from the study. It has also been stated that the maxillary sinus is further pneumatized vertically due to the loss of posterior maxillary teeth, particularly when tooth roots protrude into the sinus cavity [ 26 - 27 ] while conflicting evidence suggests reduced sinus volume following tooth loss [ 10 ]. This study excluded participants with more than one missing maxillary posterior tooth to minimize potential confounding factors. However, due to the wide age range (20-70 years), excluding subjects with single-tooth loss was deemed impractical. 

According to the results of this study, all measurements of the maxillary sinus, including sinus height, width, length, and distance between maxillary sinuses, were significantly greater in males than in females. This finding aligns with previous articles . Other studies [ 5 , 13 , 15 , 25 - 26 , 31 ] has reported similar results, although they did not include the distance between the two maxillary sinuses in their analysis. Conversely, Paknahad . [ 16 ] and Fernandes . [ 32 ] found no significant difference in sinus width between genders. However, they did observe that the height and anterior-posterior dimensions of the maxillary sinus were greater in males, consistent with our findings. Based on Ariji ., [ 11 ] the greater dimensions in the maxillary sinuses in men could be related to the greater body width and height in men compared to women.

In contrast to these studies, Saccucci . [ 14 ] disputed the notion that the maxillary sinus is a reliable sex predictor, as they observed no significant difference in mean maxillary sinus volume between males and females. They attributed this controversial finding to the distinction between volumetric and linear measurements. However, this rationale should be interpreted cautiously, given that several studies employing both linear and volumetric measurements on the same samples consistently reported larger dimensions in males compared to females [ 18 , 29 , 33 - 35 ]. 

In the present study, when age was considered, the width of the maxillary sinuses in the 30-39 age group and the length of the left maxillary sinus in the 40-49 age did not exhibit statistically significant differences between male and female subjects. However, all other measurements remained significantly higher in males compared to females. To the best of the authors’ knowledge, there are limited studies that have compared maxillary sinus dimensions/volume between the two sexes across different age groups [ 3 , 36 ]. However, these studies employed diverse age ranges within their total samples and sample classifications, which may limit the reliability of comparisons with our findings. Akhlaghi . [ 3 ] categorized their subjects into three age groups: 20-34, 35-49, and ≥50 years. They showed that while all the measurements were generally higher in males compared with females when age groups were considered, various dimensions did not show any significant difference between males and females. For example, all the sinus measurements in individuals over 50 years had no significant difference between the two sexes. Additionally, in the 35-49-year-old age group, the width of the maxillary sinuses and the right maxillary sinus's length and height did not differ between males and females. In other words, based on their results, the difference between males and females was more significant in the youngest age group (20-34 years). Aktuna Belgin . [ 36 ] analyzed maxillary sinus volumes across five age groups (18–24, 25–34, 35–44, 45–54, and ≥55 years) to compare gender differences. They showed that only males in the youngest group (18-24 years) had significantly larger maxillary sinus volume than females, aligning with observations by Akhlaghi . [ 3 ]. 

Based on the findings of the present study, dimensions of the maxillary sinuses do not change significantly in different age groups, except for the length of the maxillary sinus in females, which demonstrated statistically significant differences between the youngest (20–29 years) and oldest (≥50 years) subjects. These findings are almost in accordance with Radulesco . [ 37 ] and Sahlstrand-Johnson . [ 38 ], who reported that maxillary sinus volume remains stable throughout life, independent of age-related changes. Jun . [ 25 ] also stated that maxillary sinus volume changes significantly until maximum growth. After that, there is no correlation between the maxillary sinus volume and age. Akhlaghi . [ 3 ] also found no significant differences between age groups in female subjects. In contrast, in male subjects, almost all the dimensions of the maxillary sinuses were significantly greater in the youngest age group (20-34 years) compared to the other age groups (35-49 and ≥50 years). They found no change in the maxillary sinus dimensions compared to 35-49 and ≥50 year-old age groups. Almost similarly, Aktuna Belgin . [ 36 ] showed an inverse correlation between age and sinus volume, with significantly larger sinus volumes observed in patients aged 18–24 compared to those over 35. Velasco-Torres [ 10 ] also reported that aging reduces both linear and volumetric dimensions. These discrepancies may stem from variations in sample size, dentition status, age range, age-group classification, methodological differences, and statistical approaches employed across studies.

In this study, based on discriminant analysis, the best sex indicator among the anthropometric indices of the maxillary sinus was maxillary sinus width followed by height, the distance between sinuses, and length. Likewise, the findings of Urooge . [ 34 ] and Ahmed . [ 39 ] reported sinus width as the best sex predictor among maxillary sinus measurements. On the contrary, in some articles, the best predictor was found to be sinus height , the distance between maxillary sinuses [ 3 ], and length [ 35 ]. The variation in these findings could be attributed to differences in methodology and reference points, as well as the use of CT compared to CBCT, primarily resulting from the different acquisition techniques. While in CT, sequential slices are captured, in CBCT, the whole slices are taken by a single cone-shaped shot and then, they can be reformatted to desired cross-sections. CT images consist of predefined cross-sections with unchangeable intervals between those cuts, whereas practitioners can manually set the interval between CBCT cross-sections to smaller values. Based on Ekizoglu O. . [ 41 ], using thinner slices would lead to a higher accuracy in determining sex. Therefore, CBCT provides more precise results. 

The present study pioneered the application of discriminant analysis to identify the most robust sex predictor for each age group. Additionally, a discriminate score based on an age-specific formula and its accuracy for differentiating between two sexes was derived separately for each age group. The findings revealed that width remained the strongest sex determinant in the 30–39 and ≥50-year age groups. The 20–29-year group demonstrated superior sex prediction using the intermaxillary sinus distance, while the 40-49-year group showed maximum sinus height as the most accurate predictor. These results highlight the critical importance of incorporating age as a variable when determining sex through maxillary sinus indices. This conclusion aligns with prior research demonstrating post-skeletal maturity changes in maxillary sinus dimensions, including both volumetric increases and reductions throughout adulthood [ 10 - 12 , 42 ]. 

The only other study examining the influence of age on the accuracy of the anthropometric indices of the maxillary sinuses in sex determination was conducted by Akhlagi . [ 3 ]. However, this study failed to apply discriminant analysis, a multivariate analysis with more reliable results, and did not establish a discriminant score for specific age groups. This gap underscores the need for expanded research across diverse ethnic populations that systematically incorporate age as a critical variable when analyzing maxillary sinus dimensions for sex determination.

The accuracy rate of the sex discriminant formula was 78.3% for the 20-29 and 40-49 age groups, and 71.7% for the 30-39 and ≥50 age groups. Since this study was the first to provide an age-specific discriminant score and assess its accuracy for differentiating between sexes separately for each age group, there were no comparable studies to reference for comparison. Akhlaghi . [ 3 ] reported accuracies between 62.8% and 74.3% for right and left maxillary sinus indices in the 20-34-year age group. In the 35-49 age group, they reported the accuracies for the height (61.9%) and length (62.8%) of the left maxillary sinus since these were the only parameters in this age group that showed a significant difference between male and female subjects. Similarly, the ≥50 age group only reported an accuracy rate of the left maxillary sinus height (65.7%). They also stated that the sinus indices in individuals over 50 cannot be considered good sex identifiers. This contrasts significantly with our findings showing 71.7% accuracy in the ≥50-year age group. This discrepancy may be explained by different methodologies and statistics applied. As their reported results showed, Akhlaghi . [ 3 ] evaluated the accuracy of sex determination for each maxillary sinus index. They selected only those indices that showed significant differences between the sexes based on t-test results, which is a univariate analysis. In contrast, the present study provides one accuracy value for each age group based on the discriminant score derived from assigning weight factors to all anthropometric indices. 

Nevertheless, it is critical to emphasize that evaluating age-related changes in sinus dimensions would ideally require longitudinal studies involving repeated examinations of individuals at various life stages. However, such an approach raises ethical concerns and poses practical challenges in maintaining consistent imaging protocols over a lifelong period. Additionally, existing evidence suggests a correlation between maxillary sinus dimensions and skeletal size, particularly in transverse or anteroposterior dimensions [ 11 ]. The present study considered the transverse mid-facial skeletal size as the distance between the two maxillary sinuses. However, it is suggested that future investigations consider the zygomatic-occipital distance, body height, and weight of the subjects.

## Conclusion

Maxillary sinus measurements consistently showed larger dimensions in males compared to females across most parameters. However, this pattern was not uniform across age groups; width of the maxillary sinuses in the 30-39 age group and the length of the left maxillary sinus in the 40-49 age group displayed no statistically significant sex-based differences. Generally, the most reliable sex indicator among maxillary sinus anthropometrics was maxillary sinus width followed by height. However, discriminant analysis showed that the predictive accuracy differed by age group; for ages 20-29, the distance between the maxillary sinuses was most predictive, for ages 30-39 and 50 and above, sinus width was the strongest predictor, and for ages 40-49, the maximum sinus height was the most reliable indicator. These results highlight the importance of incorporating age as a key variable in sex determination protocols using the anthropometric indices of the maxillary sinuses. The specific sex discriminant formula presented in this study showed notable accuracy rates for Iranians; which was 78.3% for 20-29 and 40-49 years groups and 71.7% for 30-39 and ≥50 age groups.
